# Annual Meetings of Hospitals

**Published:** 1921-05-28

**Authors:** 


### ANNUAL MEETINGS OF HOSPITALS.

### St. Paul's Hospital.

Lord Dewar, presiding on May 18 at the annual meeting, said that it would be a tragedy if the hospital were allowed to "go under." Several members of the Committee of Management have personally guaranteed to the bankers the sum of £20,000, but as that limit has now been reached the work of the new hospital in Endell Street has been held up. Unless some generous friends come to the rescue he fears the work will be stopped altogether. The progressive increase in the number of patients makes the provision of larger accommodation a vital necessity. The patients' attendances in 1920 numbered 25,097, and were more than double those of 1916. The Ministry of Health has increased its grant from £1,000 to £1,500, but these grants do not cover the full expense of the V.D. treatment, and no grants are given towards capital expenditure. Owing to rebuilding operations the hospital was unable to treat in-patients during the year. The projected new building in Endell Street will contain twenty-eight beds, and an out-patient department capable of coping with over 100,000 attendances per annum. The attendances by the medical and surgical staffs will be increased to two daily sessions each for both male and female patients. There will be a resident house surgeon, and the hospital will be open day and night for the treatment of urgent cases. The total expenditure will, it is now estimated, amount to £52,000. Towards that amount £10,000 has been received.

### Royal National Orthopædic Hospital.

Lord Denbigh presided at the annual meeting held recently. The report announces the acceptance of the position of President by Prince Henry, and states that the Rt. Hon. Reginald McKenna is now joint Hon. Treasurer with Sir Samuel Scott, Bart. The number of beds for discharged and disabled soldiers has been reduced to twenty-five, and there are now 170 freed for the ordinary work of the hospital. Out-patients' attendances increased from 59,181 in 1910 to 82,253 in 1920. That increase serves to emphasise the urgent need for the extension of the present out-patients' department. The King's Fund's allocation of £24,000 to the building scheme was conditional upon an equal sum being raised by the institution before March 31. The Committee asked for an extension of the time limit. It is estimated that the building scheme will involve an expenditure of £200,000. Prince Henry will be present at a performance in aid of the fund at the Winter Garden Theatre on May 30. The patients' card collection last year amounted to £1,600, or an increase of more than £600 over 1919. The Mary Wardell Convalescent Hospital at the top of Bockley Hill, Stanmore, has been bought for £1,800, and the Committee has had to undertake to invest the sum of £2,199 as a trust to apply the income in providing convalescent treatment for women and children.

### Evelina Hospital.

The Earl of Bessborough presided at the recent annual meeting. The report draws attention to the very serious condition of the hospital finances, the deficit exceeding £3,000. The Committee is making a special effort to raise £10,000 to extinguish the deficit, and to provide the sum required to repaint entirely the hospital and carry out necessary repairs. With the object of securing new supporters a Ladies' Association has been formed, under the Presidency of the Marchioness of Carisbrooke. The annual expenditure is about £14,000, but the assured income falls far short of that sum, and about £9,000 has to be raised annually.

